# Protein activities driven by amino acid composition

**DOI:** 10.1016/j.jbc.2025.110640

**Published:** 2025-08-28

**Authors:** Sean M. Cascarina, Eric D. Ross

**Affiliations:** Department of Biochemistry and Molecular Biology, Colorado State University, Fort Collins, Colorado, USA

**Keywords:** intrinsically disordered protein, prion, stress granule, transcription, structure-function, low-complexity domain, liquid-liquid phase separation, prion-like domain, amino acid composition, biomolecular condensate

## Abstract

A foundational concept in protein biochemistry is that the primary sequence of a protein determines its structure and function. However, some protein regions defy this constraint, with protein activity dictated more by amino acid composition than primary sequence. In this review, we examine the concept of “composition-driven protein activities.” We discuss experimental criteria for classifying protein activities as driven by composition, how these criteria relate to primary sequence and composition, and how they have aided in characterizing existing examples of composition-driven activities. This emerging perspective provides a framework for thinking about the relationship between sequence and function in particular types of proteins.

The sequence→structure→function paradigm remains an important aspect of structural biology and biochemistry. For well-folded proteins, even slight changes to primary amino acid sequence can have substantial effects on their functions, and folded proteins often exhibit primary-sequence conservation across organisms to maintain activity. In contrast, intrinsically disordered regions (IDRs) do not adopt a stable 3-dimensional structure yet can still carry out important biological functions. The primary sequences of IDRs often evolve faster than structured regions and can diverge considerably while still maintaining activity (1, 2, 3, 4). Some IDRs contain short linear motifs (SLiMs) that are critical for their functions (5, 6, 7, 8). Other IDRs do not have strict primary-sequence requirements but still rely on loose primary-sequence properties such as the distribution or spacing of certain residues (9, 10, 11, 12, 13, 14, 15, 16, 17). Finally, some IDRs retain activity simply by conserving amino acid composition despite substantial primary-sequence divergence (15, 16, 18, 19, 20, 21). Thus, protein activities can be classified on a spectrum from complete dependence on primary sequence to complete independence of primary sequence.

Low-complexity domains (LCDs) are regions in proteins for which a small subset of the 20 canonical amino acids constitutes a large percentage of the sequence. Although not all LCDs are intrinsically disordered, LCDs enriched in polar and/or charged amino acids tend to be disordered (22, 23), and these types of LCDs are more common than hydrophobic LCDs (24, 25). The compositional biases that define LCDs are often a dominant determinant of their biochemical behavior. As a result, specific protein activities are statistically enriched among distinct classes of LCDs in a composition-dependent manner (15, 16, 24, 25, 26, 27, 28, 29, 30, 31, 32, 33), suggesting that the compositional biases that define LCDs may, in some cases, be key determinants of their activities.

In this review, we discuss experimental criteria for classifying protein activities as composition-driven. We then examine—through the lens of these criteria—two cases where protein activity depends more strongly on amino acid composition than on primary sequence: 1) prion formation or recruitment to stress granules (SGs) by prion domains (PrDs) and prion-like domains (PrLDs), and 2) transcription activation by acidic transactivation domains (TADs). We evaluate the evidence suggesting that these activities are primarily driven by amino acid composition and the mechanistic basis for the composition dependence. While composition-driven protein activities are apparently possible even for structured protein domains (34), we focus mostly on IDRs and LCDs, as these types of domains have thus far been more commonly linked to composition-driven activities (refer to Table 1 for a list of definitions).

**Table 1:** Terms and abbreviations

Term or Abbreviation	Definition
Primary sequence	The linear sequence of amino acids comprising a protein
Composition-driven activity	A protein activity that depends more on amino acid composition than the primary sequence
Experimental indicators	Experimental observations that support a biological hypothesis or biological model
Sequence features	The components of a protein sequence important for activity, which can include primary-sequence motifs, amino acid composition, or both
IDR	intrinsically disordered region
SLiM	short linear motif
LCD	low-complexity domain
PrD	prion domain
PrLD	prion-like domain
TAD	transcription activation domain (or transactivation domain)
DMS	deep mutational scanning
SG	stress granule
LLPS	liquid-liquid phase separation

## What is a “composition-driven protein activity”?

Composition-driven activities are those for which the amino acid composition of a protein—rather than its primary sequence—is the *predominant* intrinsic feature that determines whether a sequence encodes the activity. Composition need not be the *sole* determinant of activity: in some cases, primary-sequence elements may still contribute to activity levels (*e.g.*, by tuning activity levels). Composition may even effectively guarantee the presence of key primary-sequence elements in a protein by controlling sequence statistics through compositional bias. Therefore, amino acid composition and primary sequence are not mutually exclusive alternatives: both can contribute to protein activity even for composition-driven activities.

## Relationships between primary sequence, amino acid composition, and protein activity

**Figure 1:**
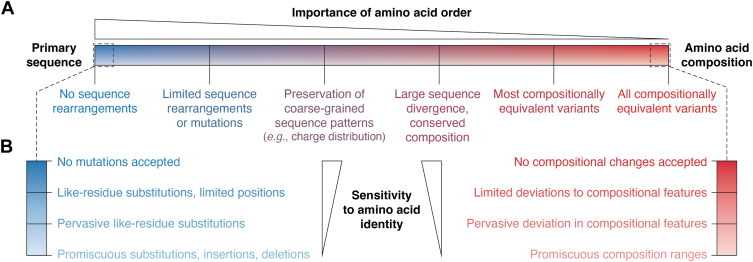
**Degrees of dependence on primary sequence and amino acid composition.** Two major axes can be used to describe constraints on the relationship between protein sequences and protein activities: the importance of amino acid order and the sensitivity to amino acid identity. *A*, protein activities can be depicted on a spectrum from complete dependence on amino acid order (*left extreme*) to complete dependence on amino acid composition (*right extreme*), where amino acid order is irrelevant for activity. Labels below the spectrum represent examples of features that may be required for a given protein to maintain activity. These examples are intentionally ordered relative to one another, but their precise positions on the spectrum are not meaningful. *B*, at each point in the spectrum in panel A (*i.e.*, when importance of amino acid order is held constant), a protein activity may exhibit different sensitivities to changes in amino acid identity. For activities mostly dependent on primary sequence, this effectively represents the tolerance to substitutions at each position. For activities mostly dependent on amino acid composition, this represents the magnitude of changes in amino acid composition that are tolerated before a given activity is lost.

The relationship between a protein’s sequence and activity can be described on two axes: sensitivity to the order of amino acids and sensitivity to changes in amino acid identity (Fig. 1). First, a purely primary sequence-dependent activity is one where any rearrangements in primary sequence impact protein activity (Fig. 1*A*, *left*), while a purely composition-driven activity is one where all compositionally equivalent variants show equivalent activity (Fig. 1*A*, *right*). Most proteins or domains lie somewhere between these two extremes. Even a structured, primary sequence-dependent protein may possess specific regions, such as linkers, that could tolerate primary-sequence rearrangement. Similarly, a domain with composition-driven activity may contain simple SLiMs or require general patterns of amino acid distribution for full activity. Second, sensitivity to amino acid identity also exists on a continuum: even proteins with a high degree of primary-sequence dependence typically have some degree of tolerance for mutations depending on the importance of the mutated region and the nature of the mutation (Fig. 1*B*, *left*). Similarly, proteins or domains whose activity is predominantly composition-dependent can have a range of tolerance for compositional changes (Fig. 1*B*, *right*), and within a specific composition-dependent domain, different compositional features may show differing sensitivity to mutation.

It is worth noting that for any given protein activity, coexisting classes of sequence-driven or composition-driven proteins could use different means to manifest that activity. For example, structured RNA-binding domains may depend on primary sequence for activity, whereas positively charged IDRs may bind RNA predominantly by virtue of their composition. These different types of domains may even coexist within a single protein.

## Composition-driven activity is often linked to IDRs and LCDs

Well-folded proteins typically depend on both the identities and positions of amino acids in the sequence to adopt specific structures and manifest their activities. In contrast, IDRs are dynamic ensembles of rapidly interconverting conformations (35). Intrinsic disorder itself is loosely considered a composition-driven activity: IDRs tend to have fewer hydrophobic amino acids, more polar amino acids, and higher net charge compared to well-folded domains, and these characteristics are even sufficient to predict disorder propensity with reasonable accuracy (22, 35, 36). Additionally, conformational ensembles differ between IDRs, and the unique properties of each ensemble can give rise to distinct behaviors and activities, leading to some degree of specificity in IDR interactions and function. These conformational ensembles can also include the formation of transient secondary structural elements, which may be influenced by binding to partner molecules. Importantly, amino acid composition often plays a key role in dictating the properties of conformational ensembles (reviewed in refs. (22, 35)). For example, a high net charge can result in expanded conformational ensembles. The propensity to form collapsed or expanded conformational ensembles depends on the propensity to interact with other residues in the IDR (intrachain interactions) relative to the propensity to interact with the solvent or surrounding molecules (22). These propensities are dictated largely by amino acid composition, although some loose primary-sequence elements such as charge distribution also affect conformational ensembles (9). Furthermore, IDRs with distinct compositions may respond differently to a variety of stimuli (*e.g.*, pH, temperature, solvent, osmotic pressure, osmolytes/electrolytes, small molecules, post-translational modification, *etc.*) by virtue of their specific compositional biases. Therefore, the relationship between compositional features and conformational ensembles of IDRs at least partially explains how composition could drive protein activity with limited contribution from primary sequence and how distinct compositions could lead to divergent activities. For a comprehensive discussion relating IDR composition to conformational preferences, see references (22, 35).

The relationship between composition and structural tendencies of IDRs raises the exciting possibility that more specific biological activities of IDRs may be encoded predominantly by amino acid composition. But it is equally important to note that this will not apply to all IDR activities, as some will also depend on the primary sequence for activity (*e.g.*, (37)). This also does not preclude the possibility that well-folded domains could exhibit composition-driven activities.

## Experimental indicators of composition-driven protein activities

**Figure 2:**
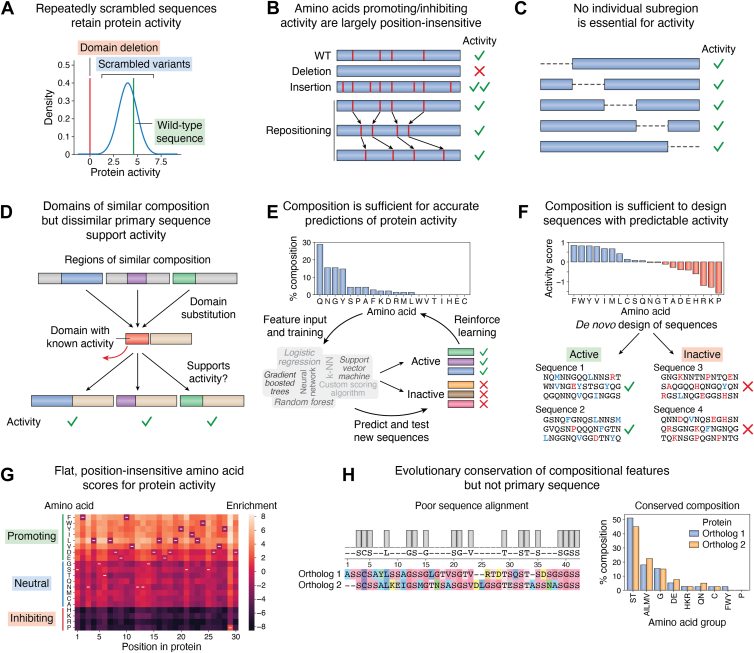
**Experimental indicators supporting composition-driven protein activity.***A*, the majority of repeatedly scrambled sequences retain detectable levels of the protein activity, though the degree of activity encoded by each scrambled variant may vary. *B*, randomly repositioning key amino acids within a sequence generally retains the same effect on protein activity (either promoting or inhibiting). *C*, scanning deletions of small fragments reveal no individual fragment that is essential for activity. *D*, replacement of a domain with sequences of similar composition (but dissimilar primary sequence) results in fusion proteins that retain the activity of the original protein. *E*, amino acid composition alone is sufficient to build or train reasonably accurate predictive models. *F*, compositional features alone can be used to design artificial sequences with or without activity. *G*, contributions of each amino acid to activity are largely position insensitive. *H*, key compositional features of sequences are evolutionarily conserved even though the corresponding primary sequences may diverge considerably.

The steady improvement of tools to predict protein structure, intermolecular docking, and intrinsic disorder allows for *in silico* analysis of the relationship between amino acid composition and characteristics such as predicted structure, interactions with binding partners, or conformational ensembles. These tools may accelerate the discovery of composition-driven activities, *e.g.*, by screening large numbers of sequence variants for activity *in silico*. However, in most cases, experimental evidence is still required to demonstrate whether the biological activity of a protein is primarily dependent on composition. A variety of experimental observations have been used to suggest that an activity is composition driven (Fig. 2). We refer to such observations as “experimental indicators” that an activity is compositionally driven, whereas protein sequence “features” refer to the specific sequence/compositional elements that drive the protein activity. In the sections below, we discuss some of the most common experimental indicators, their implications, and their limitations. While none of these experimental indicators alone serve as definitive proof of composition-driven activity, they can provide complementary support for composition-driven activity and reveal the extent to which an activity depends on composition *versus* primary sequence.

### Repeated scrambling of the sequence does not abolish activity

A hallmark of composition dependence is the maintenance of protein activity upon repeated scrambling of the protein’s primary sequence (Fig. 2*A*). In practice, it is unlikely that every scrambled variant will retain the same level of activity. However, for composition-driven activities, the distribution of activity levels for scrambled variants will tend to be higher than the activity levels when the domain is deleted entirely or replaced with a domain known to have no relevant activity. The width of the distribution indicates the degree to which the primary sequence contributes to the activity level: a larger standard deviation would suggest a greater contribution of the primary sequence. A distribution of protein activity levels may never fully separate from the activity level corresponding to the domain deletion: even for activities driven almost entirely by composition, a subset of primary-sequence arrangements may exhibit little activity.

Some IDRs have a “modular” architecture, where local subregions within the IDR possess distinct compositional and biochemical characteristics (38). This type of non-uniform distribution of composition can have functional consequences: some activities may require an evenly spaced distribution of certain features (10), whereas others may require a clustered or asymmetric distribution of specific features (11, 12, 17). For IDRs with modular architecture, scrambling can disrupt this modular pattern, potentially disrupting protein activity (38). This may necessitate careful design of scrambled variants. For example, modularity could first be spatially defined within an IDR using the Chi-Score Analysis proposed by McConnel and Parker (38). Then, modular regions could be independently scrambled, preserving both the original modularity and the relative positions of each module while disrupting any contiguous primary-sequence elements that may exist within each module.

An important caveat to sequence scrambling is that insensitivity to scrambling does not preclude the possibility that SLiMs contribute to protein activity. SLiMs are often highly degenerate segments of 3 to 10 amino acids and frequently occur in IDRs (5, 6, 7). In some cases, it is possible that SLiMs could arise by chance in scrambled variants of a sequence; this is particularly likely for LCDs, since the compositional biases inherent in the LCD may increase the likelihood of randomly generating a SLiM. Thus, targeted mutations may be needed to rule out contributions from SLiMs or requirements for broad sequence patterns.

### The levels of key residues—but not their positions—strongly affect protein activity

When dealing with IDRs and LCDs, it is tempting to assume that the most prominent compositional features are also the most important for protein activity. However, for some composition-driven activities (including the examples discussed in later sections), the most enriched amino acids merely serve as potentiators of activity rather than the main drivers of activity.

Common approaches for identifying key residues include systematic compositional changes (substitutions, insertions, or deletions of putative key features) or random mutagenesis libraries coupled with an activity assay. In domains with strong primary-sequence dependence, mutating or repositioning even a single key residue can completely abolish activity. In contrast, composition-driven activities are expected to be sensitive to the total number and density of key residues in the sequence, but only weakly sensitive to their positions in the sequence (Fig. 2*B*). Repositioning key amino acids may result in a range of activities, with the width of this distribution reflecting the extent of primary-sequence dependence. This experimental approach offers the advantage of understanding relationships between primary sequence and composition for specific residues or groups of residues independently of the rest of the sequence. A limitation of this experiment is that it requires some prior knowledge of which amino acids are most important for activity, because it would be quite labor-intensive to thoroughly characterize the position independence of each amino acid.

### No individual segment within the domain is essential for activity (scanning segment deletion)

Protein activities with strong dependence on primary sequence rely on key sequence regions for activity. While certain segments might be dispensable for activity in structured proteins (*e.g.*, unstructured loops or linkers), deletion of important segments such as stabilizing structural elements can have a strong effect on activity. In contrast, for domains with composition-driven activity, deleting any small individual segment (Fig. 2*C*) is likely to have a small effect on protein activity since the overall compositional characteristics are generally maintained for each deletion.

There are important caveats to this type of supporting data. First, some sequences may have important compositional features that are asymmetrically distributed or clustered. Thus, even for purely composition-driven activities, some variation in the impact of deletions is expected, and this variation may help reveal either key compositional features or SLiMs that promote or inhibit activity. Second, domains with composition-driven activity likely all have a minimum functional length, though this length varies for different types of domains and activities. Finally, if a sequence contains multiple instances of important primary-sequence elements distributed throughout the sequence, then scanning deletions might have little effect on activity even if that activity is driven by primary-sequence motifs.

### Replacement of a protein region with one of similar composition—but dissimilar primary sequence—recovers protein activity

If a domain’s activity is composition driven, the protein would be expected to maintain activity if the domain were replaced with one that contained the same key compositional features (Fig. 2*D*). Candidate domains can be identified based on simple compositional similarity, more targeted approaches such as searches for domains with features known to drive the activity (24, 38, 39, 40), or sequence scoring algorithms that weight each type of amino acid according to its estimated contribution to the activity (36, 41, 42, 43). Without prior knowledge of the compositional features driving activity, this approach may have a low initial success rate. However, comparison of the relative activities of candidate domains may help reveal the compositional features that are most important for activity, allowing for iterative improvement of compositional searches.

### Accurate prediction models can be developed predominantly or exclusively using compositional features

Specialized prediction models are now frequently developed to identify new proteins with a specific activity. While primary sequence is a critical input feature for many sequence-based prediction models, some protein activities are predictable using amino acid composition as the main or sole feature set (Fig. 2*E*). This experimental indicator does not preclude the possibility that predictions might improve when primary-sequence elements are incorporated into a prediction model. Indeed, for known examples of composition-driven activity, primary-sequence elements often add fine-tuning or enhancement of activity (43, 44, 45, 46).

An important caveat to this experimental indicator is that there may be some degree of crossover between primary sequence-based prediction methods and composition-based prediction methods. If different primary sequence classes are sufficiently different with respect to amino acid composition as well, then a composition-based predictor might make accurate predictions even if the activity is largely determined by primary sequence. Likewise, if the amino acid composition of a set of domains is such that a primary-sequence motif (*e.g.*, a SLiM) is likely to occur regardless of how the sequence is arranged, then a primary sequence-based predictor might make accurate predictions even if the activity is composition driven.

### Compositional features alone can be used to rationally design artificial sequences with and without a protein activity

The ability to design completely artificial protein sequences that encode the desired activity is a gold standard in validating one’s understanding of the relationship between protein features and activity (Fig. 2*F*). This typically involves construction of randomly generated primary sequences with compositional features biased toward promoting protein activity. An effective method should also be able to rationally design closely related sequences that *do not* encode the activity of interest to ensure that the prediction and design are both sensitive and specific. These designed sets of sequences would have similar compositional features but differ with respect to the key features that promote or inhibit activity. Validation of multiple designed sequence variants with similar amino acid compositions would further support a limited contribution of primary-sequence elements to the observed activity. A challenge of this experiment is that it requires extensive knowledge of the contributions of each amino acid to the activity.

### Position-insensitive amino acid scores observed in deep mutational scanning experiments

Deep mutational scanning (DMS) is a powerful approach to estimate the effect that each amino acid has on protein activity at every position in the mutagenized region (47). In DMS experiments, a region of interest is randomly mutagenized to create a large library of variants that typically deviate from the *wild-type* sequence by one or two amino acids. The library is sequenced before and after a phenotypic selection step assaying a protein activity, revealing which amino acids are more common or less common at each position after selecting for activity. For well-folded proteins, DMS can reveal positional preferences for each amino acid with remarkable sensitivity and resolution. These preferences typically appear as “hotspots” in heatmap representations of DMS data. In these cases, each amino acid is tolerable or favorable at some positions but disfavored at other positions, resulting in a bumpy, position-sensitive landscape of scores for each amino acid. In contrast, for composition-driven activities, DMS scores more strongly depend on the identities of the original and mutated amino acids rather than their position in the protein sequence. These DMS scores would appear as strips of relatively uniform color for each amino acid (though at different average levels) in a heatmap (Fig. 2*G*), revealing which amino acids are generally favored, neutral, or disfavored for the protein activity. However, in some cases, this type of experiment may not be especially informative for IDRs and LCDs as they can be less sensitive to the effects of single-amino acid changes compared to well-folded domains (48, 49). Additionally, small sample sizes for some amino acids at certain positions, as well as sampling variability, can affect enrichment/depletion estimates, which may make it difficult to identify subtle primary-sequence effects.

### Evolutionary conservation of compositional features but poor conservation of primary sequence

Proteins with composition-driven activities would be expected to maintain compositional features even if the primary sequence is poorly maintained—a phenomenon recently referred to as “compositional homology” (18). Additionally, examination of which compositional features are maintained may provide insight into the features that are most relevant for activity. For example, if the activity of a highly charged K/R-rich domain is composition driven, it might diverge substantially with respect to the positions of K and R residues yet maintain the combined percent composition of these features. The combination of these two evolutionary forces—constrained substitution with like residues and limited selection on positional preferences—could be important contributors to compositional homology, allowing domains to remain active despite primary-sequence divergence. While not an “experimental” indicator *per se*, this type of analysis can be done using common sequence alignment tools and may provide supporting or preliminary evidence of composition-driven activity.

## Experimental indicators each probe different regions of composition-versus-sequence space

**Figure 3:**
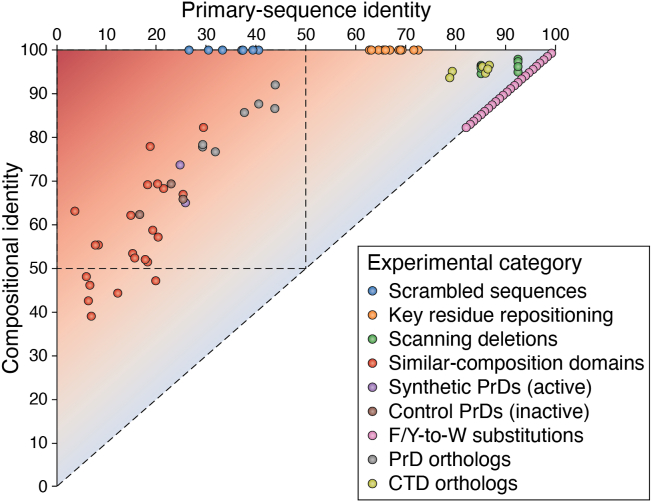
**Plot of compositional identity *versus* primary-sequence identity for experimental indicators of composition-driven activity.** Pairwise comparisons between protein sequences can be performed for both primary-sequence identity and compositional identity. Primary-sequence identity is calculated from pairwise sequence alignments using EMBOSS Needle (101, 102). Compositional identity is calculated as 100-(*d*/2), where *d* represents the Manhattan distance between the percent composition arrays for the two sequences. Each “Experimental category” corresponds to an experimental indicator from Figure 2 that suggests that a protein activity is driven by composition. The Sup35 PrD was used as a model sequence. Scrambled sequences represent seven sequences reported in a previous study (55). For key residue repositioning, the aromatic residues within the Sup35 PrD were randomly repositioned throughout the sequence for a total of 15 variants. Scanning deletions were generated by non-overlapping segment deletions of size 10 or 20 amino acids. Similar-composition domains represent the 23 unrelated prion-like domains that exhibited prion activity when substituted in place of the Sup35 PrD (53). The synthetic PrDs and control PrDs are artificial sequences that were designed to have high prion activity or no prion activity, respectively, and were tested for prion activity when substituted in place of the Sup35 PrD (59). Orthologs represent the Sup35 PrD orthologs evaluated for prion activity when substituted in place of the native PrD in *Saccharomyces cerevisiae* (19). Variants of the Sup35 PrD in which F/Y residues are progressively replaced with W residues exist on the diagonal. The Sup35 CTD is a highly conserved region that encodes a structured domain with primary sequence-dependent activity: in contrast to the Sup35 PrD orthologs, the Sup35 CTD orthologs exhibit both high compositional identity and primary-sequence identity across organisms. Deep mutational scanning sequences are not shown but would be represented as thousands of points that overlap with the upper-right *pink* dot, which represents a single F/Y to W substitution.

A major challenge in classifying activities as composition-driven or primary sequence-driven is that composition and primary sequence are not entirely separable. Most changes to the primary sequence also affect the amino acid composition, and all changes to the amino acid composition affect the primary sequence. The relationship between compositional identity and primary-sequence identity can be depicted as a two-dimensional continuum (Fig. 3). Primary-sequence identity is derived from global pairwise alignment of sequences. Compositional identity is here defined as 100 minus half of the Manhattan distance for the percent compositions of the 20 canonical amino acids. The Manhattan distance is the sum of the absolute differences in percent compositions between two sequences for the 20 canonical amino acids.

This simple calculation has a few useful properties: (1) it is on the same scale as primary-sequence identity (0–100); (2) it captures the zero-sum nature of changes in percent composition in an intuitive way (*i.e.*, as the percent composition of one amino acid goes down, others must go up by the same total degree); and (3) by definition, primary-sequence identity cannot exceed compositional identity, since all amino acids with perfect sequence identity in the primary sequence are perfect compositional matches as well. In contrast, compositional identity can exceed primary-sequence identity, since compositional features can match even if they do not align in the primary sequence. Any modifications to a protein sequence—either through experimental manipulation or evolutionary divergence—will decrease primary-sequence identity, but these same changes may affect compositional identity to varying degrees or have absolutely no effect on compositional identity.

The complementarity of the experimental indicators in Figure 2 is illustrated using the PrD of the yeast prion protein, Sup35. This protein was chosen because it is a quintessential example of a protein with composition-driven activity, and it has been thoroughly tested using almost all the experimental indicators. However, these principles are not limited to Sup35 or prion activity. While the exact effects of different experiments may be influenced by factors such as sequence complexity and the stringency of compositional requirements, the overarching principles can apply to any protein regardless of its specific activity or compositional features.

Scrambling the primary sequence greatly disrupts primary-sequence identity yet has no effect on compositional identity (Fig. 3), but the degree of primary-sequence identity for scrambled variants will depend on the sequence complexity of the original sequence. Repositioning key residues has a smaller effect on primary-sequence identity and, again, does not affect compositional identity. Scanning deletions (modeled as non-overlapping 10-amino acid or 20-amino acid deletions) result in sets of variants with identical primary-sequence identity but a range of compositional identities that depends on the relative composition of the deleted segment. Deletion of larger segments results in a greater decrease in primary-sequence identity. Compositionally similar (yet unrelated) domains that maintain activity are often shifted upward relative to the diagonal, indicating a high compositional identity but relatively low primary sequence identity. Synthetic domains designed using compositional features alone exhibit high compositional identity but relatively low primary-sequence identity. Finally, experiments such as deep mutational scanning provide valuable information on the effect of each amino acid at every position. These variants would typically appear as thousands of overlapping points in the upper right corner of Figure 3 (not shown) with minuscule changes in primary sequence identity and compositional identity. However, these overlapping points deeply probe the sequence space in that region. Additionally, sequences can exist at or near the diagonal: variants in which all F/Y residues are progressively replaced with W exhibit identical decreases in compositional identity and primary sequence identity. No W residues are present in the Sup35 PrD, so each substitution equally affects both composition and primary sequence. The degree to which identity decreases is proportional to the number of substitutions made.

In cases where compositional features are evolutionarily conserved across orthologs yet primary sequence is poorly conserved, orthologous sequences will be shifted upward from the diagonal (Fig. 3). However, even though primary sequence may be considered poorly conserved, these sequences often exhibit greater primary-sequence identity compared to the set of unrelated domains. For comparison, sequences with highly conserved, primary sequence-dependent activities (*e.g.*, the structured C-terminal domain of Sup35) exhibit both high compositional identity and high primary sequence identity (Fig. 3).

A few key features of this type of graph are worth pointing out. First, it would be impossible to achieve a sequence that simultaneously has a compositional identity of 100% and a sequence identity of 0%, so the extreme upper-left region of the graph is difficult to populate. Additionally, most known examples of sequences with composition-driven activities have strong compositional biases, and the portion of the upper-left region that is excluded will be larger for LCDs. For example, scrambled Sup35 variants still exhibit ∼30% primary sequence identity, largely due to the spurious alignments of Q and N residues, which are abundant in the Sup35 PrD.

Second, the graph highlights that similar levels of compositional identity do not guarantee that the activity will be encoded. The synthetic PrDs and control PrDs occupy a similar region in the graph (Fig. 3), yet the synthetic PrDs encode prion activity while the control PrDs do not. These differences can largely be explained by the specific ways in which the compositions deviate from those of known PrDs (41, 50). For composition-driven activities, even slight differences in the percent composition of potent activity-promoting or activity-inhibiting residues can have a large effect on activity.

Third, the graph treats each type of amino acid as distinct. In some cases, amino acids with similar physicochemical properties may be interchangeable. For example, if the activity of a D/E-rich domain is simply driven by negative charge, a D-rich domain and an E-rich domain may have very little compositional or primary-sequence identity yet possess similar activities. In other cases, amino acids with similar physicochemical properties may not be interchangeable within IDRs and LCDs (50, 51). Therefore, the choice to separate or group similar amino acids can be context- and activity-dependent. This may affect the interpretation of relationships between sequence, composition, and activity, as well as cross-study comparisons of composition-driven activities.

## Examples of composition-driven protein activities

**Figure 4:**
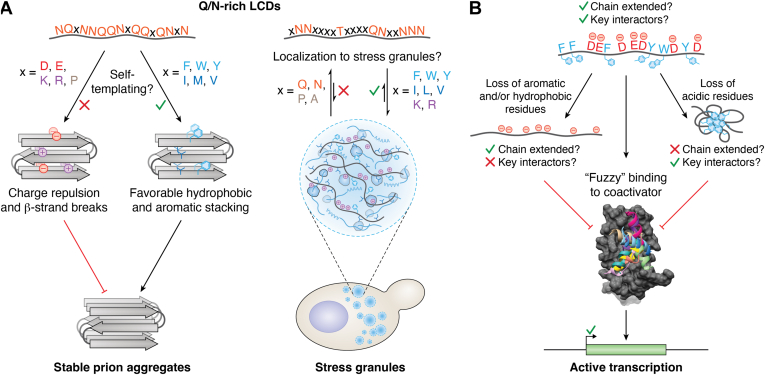
**Known examples of composition-driven protein activities.***A*, prion formation by Q/N-rich LCDs involves self-templating of parallel in-register β-sheets between identical monomers, resulting in the formation and propagation of stable amyloid aggregates (*left*). Distinct compositional features determine the degree to which PrLDs localize to stress granules (SGs) during acute stress (*right*). The example sequence represents a fragment of sPrLD2: an artificial PrLD designed to have a composition favoring SG localization (72). *B*, acidic TADs require a balance of acidic and aromatic/hydrophobic amino acids to bind to transcription coactivators and activate transcription. Loss of either acidic residues or aromatic residues limits or abolishes binding to coactivators through different mechanisms. The example sequence is based on the Pdr1 TAD from Sanborn *et al.* (44). The structural model depicts the 10 best-scoring models of a Pdr1 TAD fragment bound to the Med15 ABD1 (44), visualized using the UCSF Chimera program (103). These compositional features are also important for promoter binding specificity in some TADs (not depicted; (98, 99, 104)).

The level of experimental support for proposed or known examples of composition-driven activities varies widely. We have selected two well-studied examples of complex protein activities that are driven predominantly by amino acid composition (Fig. 4 and Table 2). These examples demonstrate some of the challenges of studying composition-driven activities and distinguishing between composition-driven effects *versus* subtle primary-sequence effects that can modulate activity.

**Table 2:** Supporting evidence for each example of composition-driven protein activity

Experimental indicator	Activities of PrDs and PrLDs		Transcription activation (acidic TADs)
	Prion aggregation	Stress granule (SG) localization	
Repeated scrambling	✓ (55, 56)	✓ (72)	✓ (44)
Position insensitivity for key residues	✓ (58)	✓ (73)	✓ (44, 88, 98)
No subregion is essential	✓ (41, 56)	?	✓ (87, 99)
Compositionally similar domains substitute	✓ (53)	✓ (72)	✓ (43, 44, 90, 92, 99)
Composition is sufficient for prediction	✓ (41, 53)	✓ (72)	✓ (43, 44, 89)
Composition is sufficient for *de novo* design	✓ (59)	✓ (72)	✓ (44, 90)
Flat distribution of position scores	?	?	✓ (97)
Only composition is well conserved	✓ (19, 20)	?	✓ (88, 98, 99)

Each category of experimental support corresponds to an experimental indicator depicted in Figure 2. Check marks (✓) represent protein activities (titles at the top) that are supported by the experimental-indicator category (titles at the left), while question marks (?) represent experimental indicators have not yet been tested for that protein activity.

### Prion formation or stress granule localization by Q/N-rich LCDs

A classic example of a composition-driven protein activity is yeast prion activity (Fig. 4*A*). Prions are protein-based elements of inheritance. Most known prions occur *via* self-templated aggregation and propagation, which allows the protein to adopt a stably inherited alternative conformation with phenotypic consequences. The first yeast prion proteins discovered, Ure2 and Sup35 (52), have Q/N-rich PrDs with relatively few charged and hydrophobic amino acids but very little primary-sequence identity. Additional compositionally similar domains have since been shown to possess prion activity, and many can substitute for the Sup35 prion domain in supporting prion activity despite little shared primary-sequence identity (53, 54). Similarly, PrDs from Sup35 orthologs in distantly related yeast species support prion activity when substituted in place of the native Sup35 PrD in *Saccharomyces cerevisiae* (19, 20). These PrD orthologs exhibit similar compositional features yet poor primary-sequence alignment.

For the Ure2 and Sup35 PrDs, all scrambled variants retained prion activity despite little primary-sequence identity (55, 56). However, these scrambled variants exhibited a range of *de novo* prion conversion frequencies, indicating that the primary sequence does have some effect on prion activity. Furthermore, prion activity was retained in scrambled variants of the Ure2 and Sup35 PrDs after systematic scanning deletion of small segments within the PrD, suggesting that no individual subregion was essential for prion activity (41, 55).

The earliest prion prediction algorithms scored protein sequences based predominantly on amino acid composition (41, 53). Subsequent algorithms trained on larger data sets further improved prediction accuracy (42, 45, 57). Surprisingly, many types of amino acids that are rare or completely absent in known PrDs (particularly hydrophobic residues) can potently enhance prion activity (58), emphasizing that accurate prediction requires not just examining the extent of compositional similarity but also the positive or negative impacts of compositional deviations. The prion-promoting activity of these hydrophobic residues is maintained even when they are repositioned (58). Mutagenesis-derived prion propensity scores were effective at predicting prion activity but only for domains first scored as IDRs (41). Prion propensity scores were sufficient to design completely artificial PrDs with predictable prion activity (59).

The mechanistic basis for both the composition dependence and the subtle effects of primary sequence is reasonably well understood for yeast prion proteins. The high Q/N content provides a disordered scaffold that is compatible with the structure of amyloid aggregates, whereas a small number of prion-promoting residues provides a strong driving force for amyloid aggregation. Prion formation involves stacking of the PrDs to form an in-register parallel β-sheet (60, 61). In this structure, the primary interactions are stacking of the corresponding residues from adjacent peptide monomers to form long β-sheets that run the length of the fiber (Fig. 4*A*). Sequence scrambling does not change these primary interactions, explaining why most scrambled variants retain prion activity (62). However, primary sequence can impact the structure in at least two ways: by facilitating placement of amino acids with low β-sheet propensity in turns or loops (63) and by impacting the ability of adjacent strands to form steric zippers (64).

Controversy still exists about the role of short sequence motifs in prion activity. The primary sequence of short peptides impacts amyloid propensity (65, 66, 67), but the sequence requirements for these motifs are broad enough that they can occur by chance even in scrambled PrDs or compositionally similar domains. Nevertheless, incorporating primary-sequence elements into prion prediction algorithms appears to modestly improve prediction performance (45, 46). These results highlight the challenges of separating composition from primary sequence and show that composition and primary sequence are not mutually exclusive alternatives even in a model that prioritizes composition.

Interestingly, while numerous Q/N-rich PrLDs exist in yeast, humans, and other eukaryotes (25, 68, 69, 70), only a small fraction of these PrLDs exhibit *bona fide* prion activity (53). Some PrLDs appear to have alternative composition-driven activities. For example, proteins with PrLDs are enriched in SGs, which are ribonucleoprotein granules that form in response to acute stress in eukaryotes and likely aid in the survival of the stress (71). PrLDs can play a role in targeting proteins to SGs and/or affecting SGs themselves (72, 73, 74, 75). Multiple experimental indicators suggest that PrLD localization to SGs can be a composition-driven activity (Table 2 and Fig. 4*B*) (72, 73). Importantly, the compositional features driving SG localization only partially overlap with those driving prion activity. While hydrophobic and aromatic residues promote both prion activity and SG localization, charged residues strongly inhibit prion activity but promote SG localization. Q and N were approximately neutral with respect to prion activity but were strongly anticorrelated with SG localization. These critical examples demonstrate that the compositional features driving distinct protein activities can diverge even for similar types of protein domains.

A mechanistic understanding of the basis for composition-driven partitioning of PrLDs into SGs is beginning to emerge. SGs are thought to form by liquid-liquid phase separation (LLPS), a process in which a solution demixes into two phases: a dense phase that is enriched in specific macromolecules and a dilute phase that is depleted of these macromolecules (76). LLPS by purified PrLDs has been described using a sticker-and-spacer model, where a small subset of amino acids engage in weak interactions that drive LLPS (“stickers”), while the intervening segments of the PrLD act as flexible “spacers” (77). Aromatic amino acids in particular have been implicated as key stickers (10, 17, 78, 79, 80, 81, 82). Since the stickers engage in non-specific interactions and the spacers provide flexibility, the saturation concentration for LLPS by PrLD-containing proteins is strongly correlated with sticker number but is largely independent of the positions of these stickers (10, 78, 79, 80). However, there are limits to this primary-sequence independence: for example, dispersion of aromatic stickers throughout the sequence promotes LLPS (10).

While the interactions driving the formation of multi-component SGs are more complicated than single-component LLPS, it is likely that similar principles apply. The primary-sequence independence observed for PrLD partitioning into SGs suggests that some combination of non-specific sticker-and-spacer interactions and/or simple solvation effects (83, 84, 85) is the predominant force driving partitioning.

Collectively, these PrDs and PrLDs demonstrate the complexity of studying composition-driven activities. It is difficult to fully separate composition from primary sequence, and even this full suite of methods may be insufficient to detect loose primary sequence requirements. Additionally, a given domain may encode multiple activities with distinct compositional requirements.

### Acidic TADs

TADs are domains within transcription factors that bind to transcription coactivator complexes (86). A substantial fraction of TADs are IDRs that are enriched in acidic residues (D/E). In addition to their canonical role in binding coactivator complexes, these acidic TADs also contribute to chromatin binding-site specificity (87). Acidic TADs typically contain aromatic (F, W, and Y) and hydrophobic (particularly L) residues that are critical for transcription activation activity (88, 89). This has led to a model where the enrichment of acidic residues and corresponding net-negative charge favors intrinsic disorder while mitigating binding to DNA itself, which would compete with binding to the coactivator (86, 89, 90). Intrachain repulsion of negatively charged residues promotes extended, disordered TAD conformations, which allows the aromatic/hydrophobic residues to remain exposed for binding to coactivators (86, 89, 90). Therefore, as with prion activity and localization of PrLDs to SGs, the most enriched features serve as potentiators for other types of residues—often present in smaller quantities within the domain—by providing a context that is intrinsically disordered (Fig. 4*C*). While the precise nature of these features will vary by activity, this pairing of potent activating residues (or even SLiMs) embedded within specific compositional contexts is a recurring theme in IDRs and LCDs (91). We focus here on acidic TADs, since composition-driven activity has been most comprehensively explored for this class.

Multiple high-throughput studies of random sequence libraries, large mutagenesis libraries, or native protein fragment (“tiling”) libraries recover diverse TAD sequences that are enriched in acidic and hydrophobic/aromatic residues (43, 44, 88, 90, 92, 93, 94, 95, 96). All of these high-throughput methods rely on fusing potential TADs to the DNA-binding domain of a known transcription factor, underscoring the modularity and interchangeability of compositionally similar TADs. Orthologous TADs can maintain activity with high conservation of compositional features but low conservation of primary sequence (88). Prediction models using only compositional features perform remarkably well (43, 44, 89), but they also improve slightly when primary-sequence features are included. Many of the identified TADs retain activity when repeatedly scrambled (44). These studies highlight that diverse sequences with key compositional features can drive transactivation activity.

The characteristic compositional features of acidic TADs were sufficient for the *de novo* design of short artificial TADs using predefined ratios of acidic and hydrophobic/aromatic residues (44, 90). For some amino acid combinations (*e.g.*, 4D + 5F), nearly all possible primary-sequence permutations exhibited transactivation activity (44). Similarly, aromatic residues in the Gcn4 TAD tended to promote activity at nearly every position in the sequence, though some positions favored activity more than others (88). Remarkably flat activity scores were reported for each amino acid from a deep mutational scan of the 263aa TAD of the CRX transcription factor (97). Furthermore, hierarchical clustering based on these activity scores results in grouping of amino acids with similar properties (97), implying that the effects of each amino acid on activity are predominantly composition-driven and position-independent.

Scanning deletions of native human TADs reveal that some TADs do not depend on any individual fragment for activity (93). Other TADs exhibit a stronger dependence on specific fragments (93), yet no detectable primary-sequence motif is common among these fragments. Rather, the only feature that is shared by nearly all sequences (96% of necessary fragments) is the presence of at least 1 F, W, Y, or L residue, implying that the segment deletions are inhibiting activity not by eliminating specific primary-sequence motifs, but by reducing the composition of key activating residues.

Transactivation activity seems to require a balance of important compositional features. In human TADs, mutating all strongly activating hydrophobic residues (F, W, Y, and L) or all acidic residues (D and E) to alanine almost universally abolished transactivation activity (93). Among protein tiles from native yeast transcription factors, TADs with the highest activity were those with a combination of greatest hydrophobicity and most negative net charge (44). Progressively mutating either aromatic or acidic residues in these TADs to alanine resulted in stepwise decreases in transactivation activity (44). However, an excess of aromatic/hydrophobic residues near active TADs decreases their activity (44). Mutations that introduce a small number of aromatic residues into the Gcn4 TAD tend to increase activity (88), but peak activity for some TADs depends on optimal levels of both acidic and hydrophobic/aromatic residues (89). Artificial sequences with only acidic or only aromatic residues fail to support activity, but mixtures of these amino acids support activity levels comparable to a native TAD (90). Therefore, even for quintessential compositional features that are associated with an activity, more is not always better.

The yeast Msn2 transcription factor has recently been explored in detail (87, 98, 99, 100). Msn2 contains an N-terminal IDR that encodes both promoter selection activity and transactivation activity. Surprisingly, only one of these activities—promoter selection activity—exhibits hallmarks of composition-driven activity. Promoter selection activity was maintained upon: (1) scanning deletion of overlapping 200aa segments in the Msn2 IDR (99); (2) replacement of the Msn2 IDR with compositionally similar orthologous IDR regions (99); and (3) repositioning of key aromatic and hydrophobic residues (98). Decreases in promoter selection activity occurred with: (1) progressive 50aa truncations of the IDR (99); (2) deletion of increasing numbers of aromatic and hydrophobic amino acids in the IDR (98); and (3) clustering of key residues in the IDR (98).

In contrast, many of the Msn2 IDR substitutions, sequence rearrangements, replacements with orthologs, truncations, and deletions that exhibited little effect on promoter selection had a strong effect on other activities encoded in the same Msn2 IDR: Med15 recruitment and transcription activity (87). This remarkable example shows that multiple activities can be encoded within overlapping regions of an IDR and that these activities can exhibit differential dependence on primary sequence and amino acid composition.

## Conclusions

There are now multiple proteins with supporting evidence for composition-driven activities. This unorthodox relationship between protein sequence and protein activity complements the standard view that primary sequence dictates protein activities and functions. A variety of experimental indicators can be used to dissect the contributions of primary sequence or amino acid composition to protein activities. Utilization of a combination of these experimental indicators has provided strong support for composition-driven activity for certain protein domains, and these indicators will continue to uncover additional examples moving forward. While composition-driven activity is not restricted to IDRs and LCDs, these types of protein regions may be more predisposed to composition-driven activities compared to well-folded regions due to inherent compositional biases and their correspondingly unique biophysical behavior. Given the pervasiveness of IDRs and LCDs, along with the compositional diversity of these types of domains, composition-driven activities are likely involved in a myriad of cellular and molecular processes.

## Conflict of interest

The authors declare that they have no conflicts of interest with the contents of this article.
